# Subcutaneous ofatumumab for remission maintenance in pediatric idiopathic nephrotic syndrome: a case series

**DOI:** 10.1093/ckj/sfag169

**Published:** 2026-05-25

**Authors:** Ning-Xun Cui, Shi-Yi Zhu, Ru-Yue Chen, Shuang Wu, Qiang Lin, Yun Zhu, Xiao-Zhong Li

**Affiliations:** Department of Nephrology and Immunology in Children’s Hospital of Soochow University, Suzhou, Jiangsu, China; Department of Nephrology and Immunology in Children’s Hospital of Soochow University, Suzhou, Jiangsu, China; Department of Nephrology and Immunology in Children’s Hospital of Soochow University, Suzhou, Jiangsu, China; Department of Nephrology and Immunology in Children’s Hospital of Soochow University, Suzhou, Jiangsu, China; Department of Nephrology and Immunology in Children’s Hospital of Soochow University, Suzhou, Jiangsu, China; Department of Nephrology and Immunology in Children’s Hospital of Soochow University, Suzhou, Jiangsu, China; Department of Nephrology and Immunology in Children’s Hospital of Soochow University, Suzhou, Jiangsu, China

**Keywords:** children, frequently-relapsing, hypersensitivity reactions, nephrotic syndrome, ofatumumab, steroid-dependent

## Abstract

**Background:**

Rituximab (RTX) is effective for pediatric frequently-relapsing or steroid-dependent nephrotic syndrome (FR/SDNS); however, some patients develop RTX resistance or severe hypersensitivity. Ofatumumab (OFA), a fully humanized anti-CD20 monoclonal antibody, represents a potential alternative, yet its use in children remains poorly documented. This study evaluates the feasibility and safety of low-dose subcutaneous (SC) OFA in children with complicated FR/SDNS who are RTX resistant or intolerant.

**Methods:**

We retrospectively analyzed five pediatric patients (median age 14.8 years) with FR/SDNS and a history of RTX resistance or hypersensitivity. Each patient received a standardized SC OFA dose of 20 mg. B-cell depletion (CD19⁺ <1%), serum IgG levels, and adverse events—graded according to the Common Terminology Criteria for Adverse Events (CTCAE)—were monitored throughout follow-up. The primary endpoint was relapse-free remission within 12 months of the last OFA dose.

**Results:**

SC OFA achieved peripheral B-cell depletion in all patients, with a median duration of 5 months. Within 12 months, 80% (4/5) of patients maintained remission. One patient with previous severe anti-CD20 hypersensitivity tolerated SC OFA with mild (Grade 2) symptoms after ibuprofen premedication. No severe infections or exacerbations of hypogammaglobulinemia were documented.

**Conclusions:**

Low-dose SC OFA is a feasible preemptive therapy for remission maintenance in pediatric FR/SDNS, particularly for those with RTX resistance or intolerance. This outpatient-compatible route may reduce hypersensitivity risks and healthcare costs. Larger prospective trials are warranted to confirm long-term efficacy.

KEY LEARNING POINTS **What was known:**

- The therapeutic benefits of rituximab (RTX) in frequently-relapsing or steroid-dependent nephrotic syndrome (FR/SDNS) are well established, its efficacy in steroid-resistant nephrotic syndrome is controversial, with response rates approximating only 50%. Recently, ofatumumab, a fully humanized anti-CD20 monoclonal antibody, has emerged as a promising alternative therapeutic option for managing FR/SDNS.

**This study adds:**

- All five patients tolerated the subcutaneous administration route, with four achieving complete remission post-treatment. Notably, one patient with previous severe hypersensitivity reactions to anti-CD20 monoclonal antibody achieved partial remission and experienced minimal allergic manifestations when pretreated with oral ibuprofen prior to ofatumumab injection.

**Potential impact:**

- Subcutaneous ofatumumab appears to be an effective and well-tolerated therapeutic option for children with FR/SDNS, particularly for those who have developed RTX resistance or hypersensitivity. This outpatient-compatible administration route eliminates the necessity for hospitalization, minimizes hypersensitivity reactions, achieves significant disease control, and potentially reduces overall healthcare costs.

## INTRODUCTION

Idiopathic nephrotic syndrome (INS) is a glomerular disorder characterized by the clinical trial of severe proteinuria, hypoalbuminemia, and edema. The annual incidence of INS is ∼1–3 cases per 100 000 children under 16 years of age [1]. Among steroid-sensitive patients, 70%–80% experience at least one relapse during follow-up, and up to 50% develop frequent relapses or become dependent on glucocorticoids to maintain remission [2]. This phenotype, referred to as complicated frequently relapsing or steroid-dependent nephrotic syndrome (FRNS/SDNS), represents a significant therapeutic challenge. Rituximab (RTX), a chimeric anti-CD20 monoclonal antibody, has demonstrated efficacy in managing complicated FRNS/SDNS and selected cases of refractory steroid-resistant nephrotic syndrome (SRNS) [3]. However, despite its clinical benefits, most patients with FRNS/SDNS eventually relapse following reconstitution of peripheral B-cell populations after RTX-induced depletion. Ofatumumab (OFA), a fully humanized anti-CD20 monoclonal antibody that targets a distinct epitope on the CD20 molecule, has emerged as a potential alternative [4]. Recent case reports and small case series suggest that OFA may induce remission in children with SDNS [5, 6], and it has been used as a substitute for RTX in patients with circulating anti-RTX antibodies [7]. Furthermore, several small case series have indicated that OFA may be more effective than chimeric anti-CD20 antibodies in multidrug-resistant nephrotic syndrome, thereby supporting further clinical investigation [4]. Emerging evidence also suggests a role for OFA in post-transplant recurrent focal segmental glomerulosclerosis [4]. However, in a single-center randomized clinical trial comparing the long-term efficacy of OFA and RTX in children and young adults with nephrotic syndrome (NS) in remission receiving maintenance therapy with prednisone and calcineurin inhibitors (CNI), OFA was not superior to RTX in achieving steroid-free and drug-free remission at 1 year, and the two treatments exhibited comparable safety profiles [8]. In adult populations, particularly in idiopathic membranous nephropathy (MN), OFA has shown efficacy in patients who are intolerant of or unresponsive to RTX [9, 10]. It has been associated with effective B-cell depletion and a lower risk of severe infusion-related or delayed hypersensitivity reactions [9]. Nevertheless, evidence supporting the use of OFA in pediatric NS remains limited. Therefore, further clinical evaluation is warranted to assess its feasibility and potential role in this population.

## MATERIALS AND METHODS

### Study design and patient selection

We retrospectively screened pediatric inpatients with NS at the Children’s Hospital of Soochow University (Suzhou, China). All patients were aged 1–18 years at diagnosis and met established diagnostic criteria, including the presence of edema, a urinary protein-to-creatinine ratio (UPCR) ≥2000 mg/g (≥200 mg/mmol), or ≥300 mg/dl, or ≥3+ protein on urine dipstick, together with hypoalbuminemia ≤2.5 g/dl (≤25 g/l), after exclusion of secondary causes such as lupus nephritis [11]. Patients were eligible for inclusion if they met at least one of the following criteria or were diagnosed with RTX resistance: (1) FRNS, defined as ≥2 relapses within 6 months after initial remission or ≥4 relapses within any 12-month period, or SDNS, defined as two consecutive relapses within 2 weeks following dose reduction or discontinuation of prednisolone [12]; (2) a documented history of significant hypersensitivity reactions to prior anti-CD20 monoclonal antibody therapy (e.g. RTX); or (3) RTX resistance. CD19^+^ B-cell counts were monitored at intervals of up to 4 months. RTX resistance was defined according to the following criteria: (a) disease recurrence or inadequate response, including relapse of NS (UPCR ≥2000 mg/g [≥200 mg/mmol], ≥300 mg/dl, or ≥3+ proteinuria) during or after RTX therapy, or failure to achieve partial or complete remission following standard RTX treatment; (b) failure of B-cell depletion or early retreatment requirement, defined as a B-cell proportion >1% or an absolute count >5 cells/µl within 1–4 weeks after RTX (weight-adjusted dosing), or the need for retreatment within ≤3 months due to poor disease control; and (c) disease progression during RTX maintenance therapy. Ultimately, five patients who met all inclusion criteria were enrolled and received subcutaneous OFA therapy between March 2023 and March 2025.

### Data collection

Comprehensive clinical and laboratory data were extracted from electronic medical records. The collected variables included baseline demographic and clinical characteristics, prior treatment history, and details of the OFA dosing regimen. Serial laboratory assessments comprised renal function parameters (serum creatinine), UPCR, and serum albumin levels, including measurements at 12 months after the final OFA dose. In addition, serial CD19^+^ B-cell proportions, serum immunoglobulin G (IgG) levels, and treatment-emergent adverse events during therapy were systematically recorded.

### Treatment protocol

OFA was administered subcutaneously at a fixed dose of 20 mg/0.4 ml via a single-dose prefilled syringe as preemptive remission maintenance therapy. Retreatment was administered when the CD19⁺ B-cell proportion exceeded 1%, provided that urinary protein was negative or the UPCR was ≤20 mg/mmol (0.2 mg/mg). All patients received oral ibuprofen (5–10 mg/kg) 30 min prior to injection.

### Outcome assessment

B-cell depletion was assessed at least 1 week after administration based on the CD19^+^ B-cell proportion relative to total lymphocytes or the absolute CD19^+^ B-cell count. B-cell depletion was defined as a CD19⁺ B-cell proportion <1% of total lymphocytes or an absolute CD19⁺ count <5 cells/µl. The primary endpoint was disease relapse within 12 months after the last OFA dose; the secondary endpoint was relapse within 24 months after the first dose. Treatment response was categorized as follows: complete remission: defined as a UPCR ≤20 mg/mmol (0.2 mg/mg) or negative/trace proteinuria on dipstick testing for at least three consecutive days; partial remission: defined as a UPCR of 20–200 mg/mmol with a serum albumin level ≥30 g/l; and treatment failure, defined as a UPCR >200 mg/mmol with persistent proteinuria.

## RESULTS

### Patient demographics and clinical characteristics

Five pediatric patients with NS were included in this study, and their demographic and clinical characteristics are summarized in Table 1. The cohort showed a male predominance, with a male-to-female ratio of 4:1. The median age at initial diagnosis was 3.7 years (range: 2.3–5.9 years), while OFA therapy was initiated at a median age of 14.8 years (range: 10.2–18.9 years). All patients received subcutaneous OFA at a standardized dose of 20 mg per injection. The median follow-up duration after the first OFA dose was 21.8 months (range: 12–35 months).

**Table 1:** Demographic and clinical characteristics of the patients.

Characteristics		Total	
		n or median	% or range
Number of patients		5	100%
Male/Female		4/1	80/20
Age at diagnosis (years)		3.7	2.3–5.9
Age at OFA introduction (years)		14.8	10.2–18.9
Age at report (years)		16.5	11–20.8
Time from diagnosis to OFA (years)		11.2	5.8–15
Previous therapy	CTC	5	100%
	Calcineurin inhibitor	4	80%
	MMF	5	100%
	ACTH	2	40%
	Anti-CD20 monoclonal antibodies (rituximab, Henlius, dabuximab)	5	100%
	Abatacept	1	20%
	IVIG	1	20%
B cell depletion before OFA (yes/no)		undepleted	100%
Time of follow-up (months)		21.8	12–35
Remission within 12 months		4	80%

OFA, ofatumumab; CTC, corticosteroids; CNI, calcineurin inhibitor; MMF, mycophenolate mofetil; ACTH, adrenocorticotropic hormone; RTX, rituximab; IVIG, intravenous immunoglobulin. ‘Undepleted’ indicates baseline B-cell status defined as CD19^+^ B cells >5 cells/µl or >1% of total lymphocytes, in accordance with the outcome assessment criteria described in the Methods section.

### Prior treatment history

Treatment histories and clinical outcomes are summarized in Table 2, while additional treatment details and biological parameters are presented in Table 3. All patients had previously received multiple immunosuppressive therapies, including corticosteroids, CNI, mycophenolate mofetil (MMF), and anti-CD20 monoclonal antibodies such as RTX. The mean CD19^+^ B-cell proportion prior to OFA administration was 5.35% (range: 3.34–9.59). In all patients, the interval between the last anti-CD20 monoclonal antibody dose and initiation of OFA exceeded 6 months. Following prior anti-CD20 therapy, three patients developed IgG levels below 4 g/L. Among these, only Patient 1 received intravenous immunoglobulin (IVIG), whereas IgG levels in the remaining two patients normalized spontaneously upon follow-up without additional intervention.

**Table 2:** Treatment and outcomes of patients.

Case	Age at diagnosis (years)	Age at OFA initiation (years)	Age at last follow up (years)	Classification	Number of anti-CD20 monoclonal antibody courses	Previous therapy	IVIG	B-cell depletion before OFA	Interval between last RTX and OFA (months)	Time to relapse after last OFA (months)	B-cell depletion (months)	Current drugs	Follow-up duration (months)
1	5.9	18.9	20.8	SDNS	16	CTC, MMF, RTX, ACTH	Yes	No	7	21	6	None	24
2	2.4	15.8	17.8	SDNS	8	CTC, CNI, MMF,CTX,RTX, ACTH	No	No	6	3	4	CTC, CNI, MMF	22
3	3.5	18.5	19.9	SDNS	3	CTC, CNI, MMF, cyclosporine A, Tripterygium wilfordii, levamisole, RTX,	No	No	6	No relapse	6	None	16
4	2.3	10.8	12.8	FRNS	8	CTC, CNI, MMF, RTX, Aba	No	No	17	31	5	CNI	35
5	4.6	10.2	11	SDNS	6	CTC, CNI, MMF, RTX,	No	No	5	No relapse	4	CNI, MMF	12

SDNS, steroid-dependent nephrotic syndrome; FRNS, frequently relapsing nephrotic syndrome; RTX, rituximab; OFA, ofatumumab; CTC, corticosteroids; CTX, cyclophosphamide; MMF, mycophenolate mofetil; CNI, calcineurin inhibitor; ACTH, adrenocorticotropic hormone; IVIG, intravenous immunoglobulin.

**Table 3:** Additional treatment details and laboratory parameters.

Case	At OFA initiation					At 12 months after OFA					
	Treatment	UPCR (mg/mg)	Alb (g/L)	Serum creatinine (μmol/L)	IgG(g/L)	Treatment	UPCR (mg/mg)	Alb (g/L)	Serum creatinine (μmol/L)	IgG(g/L)	Response
1	CTC, MMF, RTX, ACTH	<0.01	44.7	55.6	3.81	None	<0.01	46.8	54.2	7.8	Remission
2	CTC, CTX, CNI, MMF, Henlius	0.45	41.4	41.3	6.38	CTC, CNI, MMF, Henlius	0.54	33.5	69.6	5.51	No remission
3	CNI, MMF, dabuximab	1	47	28.7	4.16	No	<0.01	47.3	37	4.88	Remission
4	CTC, CNI, MMF, RTX, Aba	0.22	34.5	29.1	6.1	CNI	0.01	42.1	37.8	9.32	Remission
5	CTC, CNI, MMF, RTX	0.21	43.1	33.2	5.68	CNI	<0.01	41.4	34.6	8.95	Remission

NS, nephrotic syndrome; OFA, ofatumumab; UPCR, urine protein-to-creatinine ratio; Alb, albumin; RTX, rituximab; CTC, corticosteroids; CTX, cyclophosphamide; CNI, calcineurin inhibitor; MMF, mycophenolate mofetil; ACTH, adrenocorticotropic hormone; IVIG, intravenous immunoglobulin; Aba, abatacept.

### Renal function parameters

Serum creatinine levels remained within age-appropriate reference ranges throughout the treatment and follow-up period for all patients, indicating preserved renal function during OFA therapy.

### Treatment efficacy and immunological response

**Figure 1:**
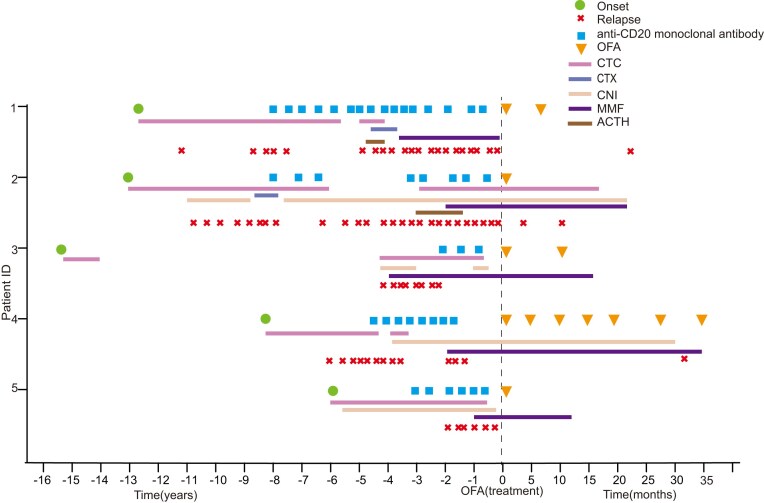
Disease course and treatment regimens before and after initiation of OFA (time 0) in pediatric patients with idiopathic nephrotic syndrome. Source: this figure was created by the authors. CTC, corticosteroids; CTX, cyclophosphamide; CNI, calcineurin inhibitor; MMF, mycophenolate mofetil; OFA, ofatumumab; ACTH, adrenocorticotropic hormone.

**Figure 2:**
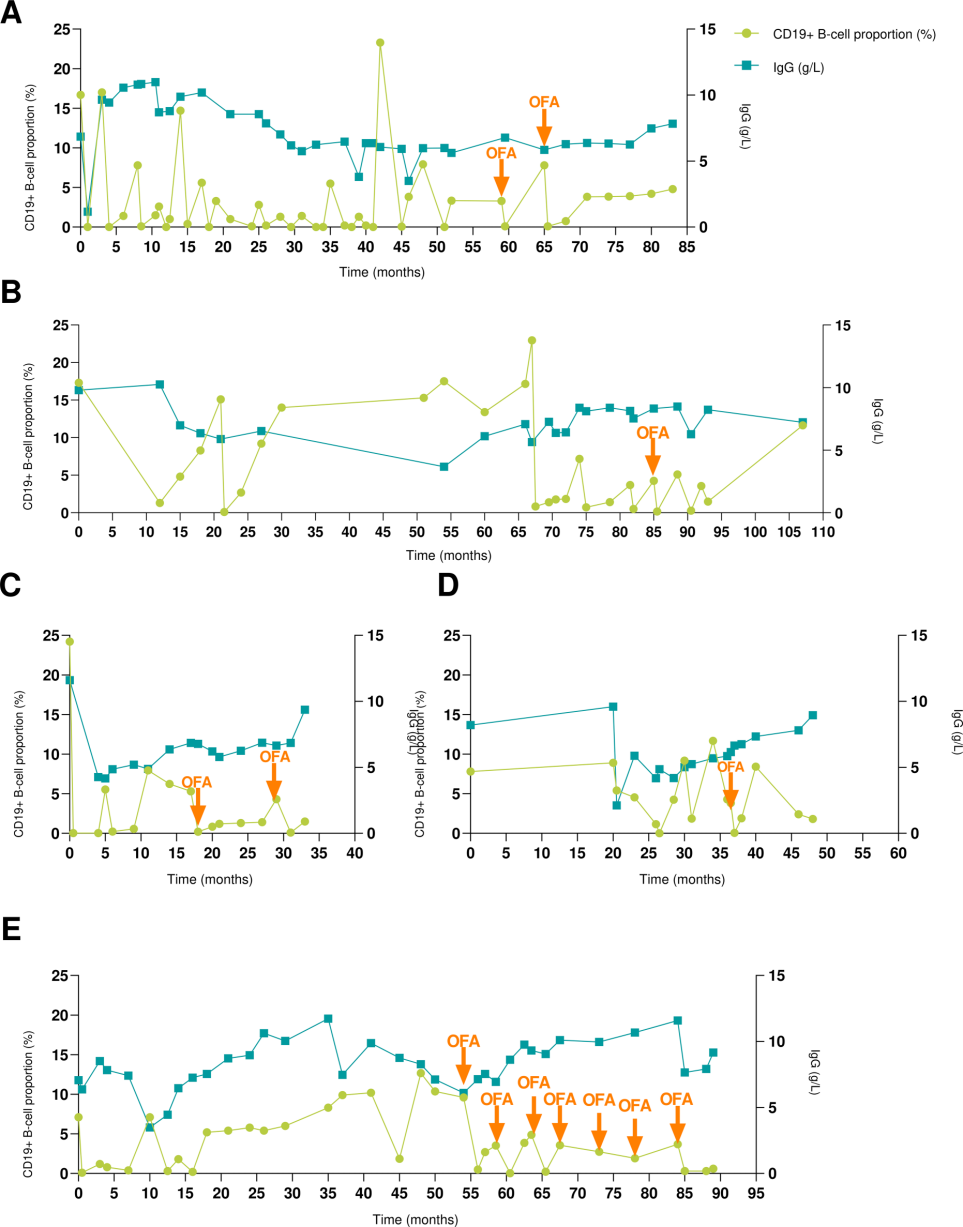
Temporal changes in CD19^+^ B-cell proportion, serum IgG levels, and OFA administration in pediatric patients, using the first RTX administration as the starting time point, with OFA dosing indicated by arrows. Panels A–E correspond to Patient 1, Patient 2, Patient 3, Patient 5, and Patient 4, respectively.

The disease course and treatment regimens before and after initiation of OFA (time 0) are illustrated in Fig. 1, and longitudinal changes in CD19^+^ B-cell proportions, IgG levels, and OFA administration are shown in Fig. 2. During the follow-up period, Patients 1 and 3 received two doses of OFA, Patients 2 and 5 received one dose, and Patient 4 received six doses. Sustained CD19⁺ B-cell depletion was achieved in all patients post-OFA, with serum IgG consistently maintained above 4 g/L during follow-up. Disease relapse occurred in Patient 2 at 3 months after OFA; proteinuria resolved after intensified treatment with corticosteroids, CNI and MMF. Four patients maintained sustained negative urinary protein throughout the 12-month follow-up. Relapse was documented in Patients 1 and 4 until 12 months post-OFA. Although OFA induced short-term disease remission in this cohort, the small sample size precluded any conclusion of superiority over conventional anti-CD20 regimens.

### Safety profile

No infections were documented in any of the five patients during the follow-up period after OFA administration. Serum IgG levels remained above 4 g/l in all patients at the last follow-up (individual IgG values at baseline and 12 months post-OFA are presented in Table 3). Three patients had a history of RTX-associated hypogammaglobulinemia (IgG < 4 g/L); IgG levels had normalized prior to OFA initiation in two of these patients without intervention, while one required IVIG supplementation. No further decline in IgG levels was observed following OFA administration.

Median B-cell depletion lasted 5 months (range 4–6 months), with reconstitution (≥1%) at a median of 3 months after the last dose. Vaccination status was not systematically documented in this retrospective study,which represents a limitation. 

### Hypersensitivity reactions

A hypersensitivity reaction occurred in Patient 4 during the initial OFA administration and was classified as Grade 2 according to the Common Terminology Criteria for Adverse Events (CTCAE) [13]. The patient received a prophylactic oral dose of ibuprofen prior to the subcutaneous injection. Within 2 h after administration, the patient developed chills, headache, and nausea. Vital signs remained stable, with normal blood pressure and a body temperature of 37.8°C. The symptoms resolved following an additional oral dose of ibuprofen, and no further adverse events occurred during the remainder of the treatment course. The other patients received a single prophylactic oral dose of ibuprofen (5–10 mg/kg) prior to injection, and no hypersensitivity reactions were observed during follow-up.

## DISCUSSION

To our knowledge, this study provides one of the earliest preliminary reports on subcutaneous OFA administration in pediatric NS. In this case series of five children with complicated NS who had failed multiple immunosuppressive therapies, including RTX, a fixed subcutaneous dose of 20 mg OFA was used. This approach differs substantially from previously reported intravenous regimens (300–2000 mg/1.73 m^2^), not only in dose but also in pharmacokinetic profile. In this case series, SC OFA was associated with maintained remission in most patients during follow-up, with generally manageable tolerability and a limited number of adverse events. Notably, patients with prior severe hypersensitivity reactions to anti-CD20 monoclonal antibodies tolerated subcutaneous OFA with only mild symptoms when premedicated with oral ibuprofen. Subcutaneous administration may offer practical advantages, including feasibility in the outpatient setting and reduced treatment burden. It may therefore represent a possible exploratory alternative for selected patients with RTX-resistant or RTX-intolerant pediatric NS. However, given the small sample size and retrospective design, these findings should be interpreted with caution and require confirmation in larger prospective studies.

Recently, B-cell-depleting agents have emerged as effective treatments for MCNS [14]. With advances in the understanding of B-cell functional subsets, an increasing number of studies have highlighted the role of B cells in the pathogenesis of INS [15]. Anti-CD20 monoclonal antibodies are effective in inducing and maintaining prolonged remission in children with INS [16]. B lymphocytes contribute to disease development through multiple mechanisms that can be broadly categorized as antibody-dependent and antibody-independent pathways. Antibody-dependent mechanisms involve dysregulated immunoglobulin production, in which specific autoantibodies may exert direct effects on podocytes. In contrast, antibody-independent mechanisms include complex interactions between T and B lymphocytes and the cytokines they produce.

Regulatory T cells (Tregs) play a critical role in maintaining immune homeostasis by suppressing excessive activation of effector T cells (Teffs). Patients with INS exhibit an imbalance characterized by reduced Treg/Th17 and Th1/Th2 ratios, along with increased expression of T helper (Th)-associated cytokines, which can directly injure podocytes and lead to proteinuria. Among B-cell subsets, regulatory B cells (Bregs) and effector B cells (Beffs) have distinct immunomodulatory functions, secreting cytokines such as IL-10, IL-6, and IFN-γ, thereby exerting either protective or pathogenic effects. In addition, factors that regulate B-cell proliferation and survival, including B-cell-activating factor (BAFF) and follicular helper T cells (TFH), may further contribute to the pathogenesis of INS [17].

Four anti-CD20 monoclonal antibodies are currently available for the treatment of NS: RTX, OFA, obinutuzumab, and ublituximab. Although these agents target the same CD20 antigen, they differ in their molecular structure and pharmacological properties. RTX is a chimeric human–mouse monoclonal antibody directed against the CD20 antigen expressed on pre-B and mature B cells [18] and is recommended by the Kidney Disease: Improving Global Outcomes (KDIGO) guidelines as a steroid-sparing therapy, particularly for pediatric patients with SDNS/FRNS [19].

RTX binds to amino acid residues 168–175 on the large extracellular loop of CD20 and induces B-cell depletion through multiple mechanisms, including complement-dependent cytotoxicity, Fcγ receptor-mediated and complement-dependent phagocytosis, and direct natural killer cell-mediated cytotoxicity [20]. In addition to its B-cell-depleting effects, RTX has been shown to bind directly to sphingomyelin phosphodiesterase acid-like 3b on podocytes, thereby stabilizing the cytoskeleton and exerting direct antiproteinuric effects [20].

However, emerging evidence suggests that repeated RTX courses may significantly increase the incidence of hypogammaglobulinemia, defined as a serum IgG level below two standard deviations of the age-adjusted mean (0–2 years: IgG < 350 mg/dl; 2–6 years: IgG < 400 mg/dl; >6 years: IgG < 500 mg/dl) [21, 22]. In some patients, this may lead to serious complications, including severe infections and neutropenia. OFA is a fully humanized IgG1κ anti-CD20 monoclonal antibody, and several case studies have reported its ability to induce remission in children with SRNS who are unresponsive to RTX [23]. The clinical application of OFA in pediatric NS was first reported by Basu *et al*. in 2014 [24]. In that study, five children with SRNS received OFA at an initial dose of 300 mg/1.73 m^2^ body surface area, followed by 2000 mg/1.73 m^2^ administered weekly for five consecutive infusions, with remission achieved after the fourth dose in three patients and after the sixth dose in the remaining patient. Subsequently, Wang *et al*. applied a similar protocol in five pediatric patients with SRNS in 2017 [6]. In contrast to these intravenous regimens, the present study provides preliminary evidence supporting the feasibility of low-dose subcutaneous OFA (20 mg) in a distinct pediatric population. The drug was administered as a 20 mg/0.4 ml solution via a single-dose prefilled Sensoready® pen (Novartis, Basel, Switzerland). The dosing strategy was initially informed by the first three patients, whose age and body weight approached those of adults, thereby supporting the use of an adult dosing regimen. For the fourth and fifth patients (aged 10.8 and 10.2 years, respectively), the dosing approach was further guided by the study by Wu *et al*. [25], in which pediatric patients were treated according to the adult multiple sclerosis regimen. In that study, three patients experienced no adverse events, while one developed transient fever and scalp pruritus during the first subcutaneous injection, followed by alopecia in the third week. Pharmacokinetic and pharmacodynamic studies of anti-CD20 monoclonal antibodies in multiple sclerosis have shown that OFA-induced B-cell depletion is largely independent of body weight, with sustained depletion observed across a broad range of patient characteristics [26, 27]. Consistent with these findings, analyses from the ASCLEPIOS I and II trials demonstrated no clear association between body weight variability and treatment efficacy, particularly with respect to progression independent of relapse activity [28]. These data provide preliminary supportive evidence for the rationale and potential safety of the dosing and administration strategy employed in this study.

Unlike earlier protocols targeting SRNS with high-dose intravenous infusions, our cohort consisted exclusively of children with complicated FR/SDNS, a phenotype characterized by recurrent relapses despite treatment with CNI, MMF, and prior RTX. This distinction is clinically important. OFA binds to discontinuous sequences within both the small (amino acid residues 74–80) and large (amino acid residues 145–161) extracellular loops of the CD20 antigen via its Fab domain, targeting an epitope distinct from that of RTX, while mediating its immune effects through the Fc domain [29]. This epitope is located closer to the cell membrane and provides a broader binding interface compared with other anti-CD20 antibodies. In addition, OFA contains a C1q binding site that enhances complement-dependent cytotoxicity [30], which may permit effective B-cell depletion at lower doses [31]. Previous studies have explored reduced-dose strategies. For example, Bonanni *et al*. treated six children with similar clinical characteristics using a “low-dose” regimen (300 mg followed by 700 mg/1.73 m^2^ over two weeks) and reported remission of proteinuria in two cases [32]. In this small case series, our subcutaneous approach was associated with remission maintenance in four of five patients within 12 months at a substantially lower dose. However, direct comparisons are limited by differences in patient populations, disease subtypes, and study designs. These findings raise the hypothesis that route-dependent pharmacokinetic differences may influence therapeutic outcomes, although this requires confirmation in prospective comparative studies. Preliminary evidence further suggests that dosing alone may not determine therapeutic efficacy; rather, route-dependent pharmacokinetic (PK) profiles may influence B-cell depletion in FR/SDNS by maintaining sustained drug exposure while avoiding high peak concentrations. Subcutaneous administration is characterized by prolonged absorption, resulting in sustained systemic exposure, reduced peak concentrations (Cmax), and a potentially lower risk of hypersensitivity reactions. However, the causal relationship between these PK properties and clinical outcomes requires validation in large prospective studies. Notably, subcutaneous OFA exhibits a relatively slow absorption rate, with peak concentrations typically reached 3–7 days after injection. Compared with previous studies, our patients demonstrated several distinct features, including multidrug resistance, prior anti-CD20 hypersensitivity, and prolonged disease duration (median 11.2 years). Three observations may help explain the clinical relevance of this protocol. First, with respect to B-cell kinetics, rapid peripheral B-cell depletion was observed within 1 week, similar to intravenous regimens; however, the median duration of depletion (5 months) appeared longer than that typically reported for RTX (2–3 months), which may contribute to delayed relapse. This difference may be related to the higher binding affinity of OFA to the cell membrane and its slower dissociation from CD20 compared with RTX [33, 34]. From a pharmacokinetic perspective, intravenous administration results in immediate systemic availability and efficient depletion of CD20-positive B cells, particularly within the spleen, due to the permeability of splenic capillaries [33]. In contrast, subcutaneous administration involves transport from the hypodermis to the lymphatic system prior to entry into the systemic circulation [35]. Although this slower absorption may reduce overall bioavailability compared with intravenous delivery, it may also mitigate adverse effects associated with high serum concentrations. In addition, the sustained absorption profile may enhance lymphoid tissue exposure and potentially disrupt memory B-cell survival niches. Second, regarding hypersensitivity mitigation, Patient 4, who had a history of severe RTX-related hypersensitivity reactions, tolerated subcutaneous OFA with only mild symptoms following ibuprofen premedication. The potential mechanisms underlying this protective effect are multifactorial. As a non-steroidal anti-inflammatory drug, ibuprofen inhibits cyclooxygenase activity, thereby reducing prostaglandin synthesis and attenuating inflammatory responses associated with B-cell lysis and cytokine release during OFA administration. In addition, its antipyretic and analgesic properties may help prevent or alleviate infusion-related symptoms such as fever, headache, and myalgia. These findings suggest that ibuprofen may be a practical premedication option for OFA administration. For patients at higher risk, combination with glucocorticoids and antihistamines may further reduce the incidence of infusion-related reactions, thereby improving treatment safety and tolerability. Third, with respect to IgG dynamics, subcutaneous OFA did not appear to exacerbate IgG depletion in this small cohort, despite prior RTX-associated hypogammaglobulinemia (IgG < 4 g/l in 3/5 patients). This observation suggests a potentially more favorable immunomodulatory profile. Although current international guidelines (KDIGO 2021) recommend RTX as the preferred therapy for children with FR/SDNS [19], our findings indicate that subcutaneous OFA may be considered as an alternative option in selected patients. This may be particularly relevant in clinical scenarios where hypersensitivity reactions limit the use of intravenous anti-CD20 therapies or where frequent RTX administration (e.g. at intervals <6 months) suggests pharmacodynamic resistance.

Although previous studies have explored OFA in pediatric NS, this study represents, to our knowledge, one of the first exploratory assessments of the efficacy and safety when administered as a fixed-dose subcutaneous regimen in children with FR/SDNS, including those with RTX hypersensitivity or requiring frequent RTX retreatment. These features, together with the prolonged disease duration observed in our cohort, may enhance the clinical relevance of our findings. However, several important limitations should be acknowledged. As a retrospective study, it is subject to missing or incomplete data, as well as potential selection and recall bias. In addition, the small sample size limits the generalizability of the results. Anti-RTX antibody testing was not performed due to methodological constraints, including the lack of access to standardized and validated commercial assays during the study period and the absence of a universally accepted quantitative reference standard, which could introduce variability in interpretation. Future studies using standardized detection methods are needed to clarify the impact of anti-RTX antibodies on treatment outcomes in this population. Furthermore, loss to follow-up limited the frequency of longitudinal assessments of B-cell dynamics after OFA administration, restricting analysis of the relationship between B-cell depletion or reconstitution and clinical outcomes. The relatively short follow-up duration in some patients (three of five with <24 months of follow-up) also precludes conclusions regarding long-term efficacy, and only short-term remission maintenance can be described. Importantly, as this study was designed primarily to assess feasibility and safety, no definitive clinical recommendations can be drawn. Nevertheless, this study provides proof of concept for outpatient-compatible B-cell depletion using subcutaneous OFA. Future prospective studies should compare subcutaneous and intravenous OFA in terms of pharmacokinetics and pharmacodynamics and explore potential biomarkers (e.g. CD19^+^ B-cell subpopulations and urinary CD20^+^ extracellular vesicles) to optimize patient selection and treatment strategies.

## CONCLUSION

In conclusion, this study represents, to our knowledge, one of the earliest reports of subcutaneous OFA administration in pediatric NS. All five patients tolerated the subcutaneous regimen well, with remission maintained during the follow-up period, a low incidence of relapse, and fewer hypersensitivity reactions than reported with intravenous anti-CD20 regimens. In addition, this approach may reduce the frequency of anti-CD20 monoclonal antibody administration, although no advantage in remission induction was evident. These preliminary findings support the feasibility and safety of subcutaneous OFA; however, given the small sample size and retrospective design, no definitive clinical recommendations can be drawn. Larger prospective studies are warranted to confirm these observations and to define the role of this approach in clinical practice.

## Data Availability

The data underlying this article cannot be shared publicly due to patient privacy and ethical restrictions. De-identified data may be made available from the corresponding author upon reasonable request and with appropriate approval.
